# Leveraging large genomic datasets to illuminate the pathobiology of autism spectrum disorders

**DOI:** 10.1038/s41386-020-0768-y

**Published:** 2020-07-15

**Authors:** Veronica B. Searles Quick, Belinda Wang, Matthew W. State

**Affiliations:** Department of Psychiatry and Behavioral Sciences, UCSF Weill Institute for Neurosciences, University of California, San Francisco, San Francisco, CA 94143 USA

**Keywords:** Risk factors, Neurodevelopmental disorders

## Abstract

“Big data” approaches in the form of large-scale human genomic studies have led to striking advances in autism spectrum disorder (ASD) genetics. Similar to many other psychiatric syndromes, advances in genotyping technology, allowing for inexpensive genome-wide assays, has confirmed the contribution of polygenic inheritance involving common alleles of small effect, a handful of which have now been definitively identified. However, the past decade of gene discovery in ASD has been most notable for the application, in large family-based cohorts, of high-density microarray studies of submicroscopic chromosomal structure as well as high-throughput DNA sequencing—leading to the identification of an increasingly long list of risk regions and genes disrupted by rare, de novo germline mutations of large effect. This genomic architecture offers particular advantages for the illumination of biological mechanisms but also presents distinctive challenges. While the tremendous locus heterogeneity and functional pleiotropy associated with the more than 100 identified ASD-risk genes and regions is daunting, a growing armamentarium of comprehensive, large, foundational -omics databases, across species and capturing developmental trajectories, are increasingly contributing to a deeper understanding of ASD pathology.

## Introduction

Autism spectrum disorder (ASD) refers to a group of neurodevelopmental disorders (NDDs) defined by social communication deficits and restricted, repetitive patterns of behavior or interests [1]. The prevalence of the syndrome is currently estimated to be ~1.7% in the US population [2]. It has been known for many decades that genetic risk plays a critical role in ASD etiology [3]. However, only in the past dozen or so years has the systematic and reliable identification of specific genes conferring liability for ASD-gained real traction. This transformation has been the result of “shared big data”, made possible by the sequencing of the human genome, advent of high-density microarrays, development of high-throughput sequencing technologies, application of rigorous statistical methods that account for vast numbers of comparisons, availability of increasingly comprehensive neurobiological databases, and prioritization of rapid data-sharing and large-scale collaborations across research groups.

**Fig. 1:**
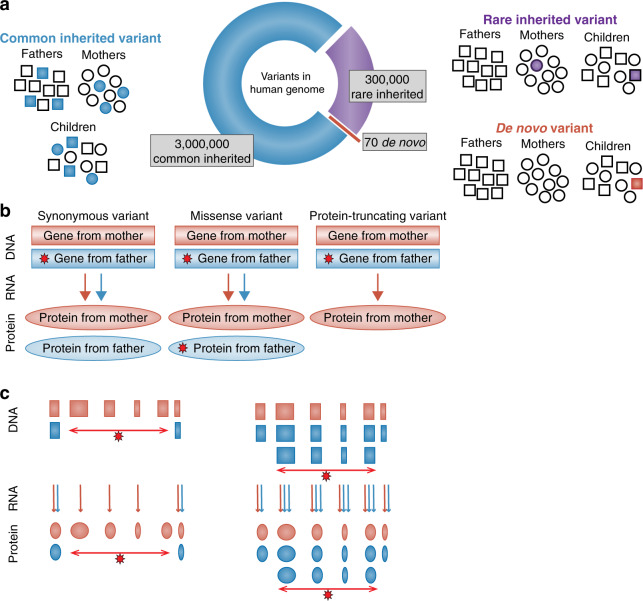
Types of genetic variants. **a** The majority of genetic variation in the human genome is common (population frequency ≥ 1%, blue). These variants are transmitted from parents to offspring via Mendelian inheritance patterns. A smaller proportion is rare (≤1%, purple) and also transmitted from parents. ∼70 variants are de novo (red), observed only in the child, but not in either parent. **b** The impact of single-nucleotide variants (SNVs) and small (≤50 bp) insertion/deletions (indels) depends on their location in the genome. In the 1.5% of the genome that encodes proteins (the exome), these variants can either be synonymous (no change to the resulting protein), missense (a single amino acid is changed in the protein with variable functional impact), or protein-truncating (leads to nonsense-mediated decay and no protein). Variants and their consequences (red stars) are shown on the father’s allele, but can also arise on the maternal allele. **c** Copy number variants (CNVs) are large (≥50 bp to millions of nucleotides) deletions (resulting in no protein), or duplications (potentially resulting in excess protein). Figure adapted from Sanders [81] with author permission.

**Fig. 2:**
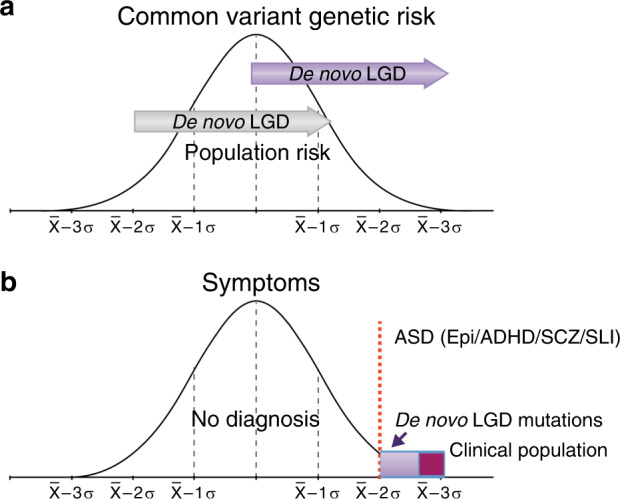
A model of rare large-effect de novo mutations acting in combination with common risk alleles. **a** An idealized distribution of common polygenic risks that are normally distributed in the general population. The red vertical dotted line represents an arbitrary cutoff for the diagnosis of ASD. For a highly heritable disorder such as ASD, those at the low end of the distribution of risk (left) will be less likely to meet diagnostic criteria than those on the far right end of the distribution. The superimposition of the upper panel and the lower panel (**b**), representing the distribution of ASD symptoms in the population, models the observation that the vast majority of common allele population risk is present in individuals without a clinical diagnosis. The lower panel (**b**) shows the same red dotted vertical line reflecting an arbitrary cutoff for the categorical diagnosis of ASD. The abbreviations in parenthesis (epi epilepsy, ADHD attention deficit hyperactivity disorder, SCZ schizophrenia, SLI specific language impairment) reflects the observation that highly penetrant ASD risks may also carry risks for diagnoses apart from ASD. The arrows on the bottom of the diagram represent large-effect rare de novo mutations. The purple arrow is showing how a large risk de novo mutation can move an individual with intermediate risk and the likelihood of no symptoms across the diagnostic threshold. The gray arrow reflects the observation that these risks while large are not Mendelian and that sometimes rare large-effect mutations do not show a phenotype at all, which may reflect that they are acting in the context of very low polygenic risk. The purple box on the right side of (**b**), reflects the finding that while de novo mutations carry a very small proportion of population risk, they represent a substantial fraction of individuals who exceed clinical thresholds.

A key outcome of this research has been the identification of a large and growing number of genes and regions that definitively confer ASD risk [4, 5] and the illumination of the overall genetic architecture of the syndrome—that is the type and relative distribution of genetic variation underlying the disorder [6] (Fig. 1). Overall, the past decade of research has confirmed that alleles that are common in the typically developing population exert very small individual effects, yet carry the majority of population risk [7]. However, among the ASD clinical population, a substantial contribution from rare, typically de novo variants of large effect have also been identified [8, 9] (Fig. 2), which offer distinctive opportunities and challenges in the pursuit of underlying biological mechanisms.

The identification of more than 100 large-effect risk genes and genomic regions has already led to important insights into molecular mechanisms. From the earliest results of reliable gene discovery efforts, synaptic structure and function and chromatin modification have been identified as the most common points of functional convergence [4, 5, 10–12]. Nonetheless, there remain important obstacles to leveraging gene discovery to develop an actionable understanding of ASD pathophysiology.

A key issue is differentiating disease mechanisms from the highly pleiotropic biology encoded by ASD-risk genes. It is more straightforward to demonstrate a range of biological consequences of a given risk mutation (e.g., differential gene expression, changes in electrophysiological properties, alterations in cellular proliferation, differentiation, or migration), than it is to confirm that a particular observation is a contributor to the human disorder. A systems biology approach to defining salient aspects of ASD pathology involves using large datasets to identify the intersection or convergence among multiple risk genes somewhere along the path from genes to behavior—for instance in relation to molecular pathways, cell types, anatomical regions, and/or developmental stages. Increasingly, -omics approaches have been employed in this effort, empowered by a growing armamentarium of large-scale foundational databases characterizing region- and cell-specific transcriptional activity across species, developmental patterns of gene expression and regulation including in humans, and proteomic pathways [13]. The rationale for and challenges facing these complementary approaches to elaborating pathophysiology will be discussed after a review of the current state of gene discovery in ASD.

## ASD gene discovery

A confluence of factors has led to the maturation of the field of ASD genetics: the sequencing of the human genome; associated rapid advances in -omics technologies, a focus on studying rare and de novo mutations as well as common alleles, early successes of parent advocacy groups in promoting the creation of large-scale open genetic resources [14], and highly effective and now long-standing partnerships among advocacy groups, academia, the National Institutes of Health and philanthropy promoting open data sharing and large-scale scientific collaborations.

### Mendelian forms of ASD

High-throughput -omics technologies and associated analytic approaches have been critical to the emergence of systematic, reliable ASD gene discovery, particularly among individuals with common forms of the syndrome. Nonetheless, the first insights into the genetic architecture and biology of social disability can be traced to studies of well-described genetic syndromes that pre-date the -omics era. These paradigmatic disorders, including Fragile X syndrome (FXS) [15–17], Rett syndrome [18], and tuberous sclerosis complex (TSC) [19, 20], provided the earliest opportunities for gene discovery largely as a consequence of an extremely high correlation between variation at a single genetic locus and a distinctive, reliable phenotype—often characterized by intellectual disability, neurological findings and/or dysmorphology, as well as an increased risk for social impairment.

As early as the 1990s, the study of these “monogenic” forms of ASD began to reveal themes that foreshadowed results of future research examining larger ASD populations. These include the importance of rare mutations, the involvement of both coding as well as noncoding variation, the finding that a highly diverse array of molecules can all lead to an ASD phenotype, often coincident with intellectual disability, and the illumination of implicated biological functions, including RNA binding, synaptic structure and function, the mammalian target of the rapamycin (mTOR) pathway, and gene regulation, epigenetics, and chromatin modification [21].

However, before focusing on the role of big data in ASD gene discovery, the above distinction between “syndromic” or “Mendelian” forms of ASD versus “common”, “non-syndromic”, or “complex forms” of the disorder warrants further discussion. These terms are often used interchangeably to differentiate a small subgroup of affected individuals who carry single mutations of large effect and have ASD in the context of other cardinal features, from the vast majority of individuals who present primarily or exclusively with the ASD behavioral phenotype. Prior to the current era of successful gene discovery, the conventional wisdom held that this latter group likely reflected complex polygenic inheritance.

While this language remains commonplace, the distinctions have not held up well to emerging the data. As described in detail below, gene discovery in ASD has identified major contributions from both common additive small-effect alleles as well as rare mutations of large effect. At present, there is no reliable way to distinguish between these based on phenotype: cohorts defined as “non-syndromic” based on observable features yield results across the full spectrum of genetic variation. For this reason, assigning individuals as “monogenic” or “simple” versus “common“ or “complex” can only be done through genetic testing. And even here, the boundaries remain porous, as the risk architecture of ASD is a continuum, making a definitive separation between complex and simple genetics the subject of ongoing debate. For example, many ASD-associated rare large-effect variants nonetheless show incomplete penetrance and variable expressivity, likely as a result of additional factors (genetic, epigenetic, or environmental) (reviewed in ref. [22]). Similarly, it has been demonstrated that common variant risk contributes to ASD liability, even in ASD individuals carrying a strongly deleterious de novo mutation [9, 23, 24].

The terms “syndromic” versus “non-syndromic” ASD fare slightly better under careful scrutiny, and we will continue to rely on them below—with appropriate caveats. For example, it is not uncommon to find rare examples of syndromic mutations in carefully done studies of individuals with ASD who are thought not to have dysmorphology or characteristic features on exam. Conversely, when overtly nonsyndromic cohorts are re-categorized based on genetic results, syndromic features may become apparent retrospectively. For example, increased head circumference has been identified as a common feature of individuals with mutations in *CHD8* [25]—a gene first identified as a highly penetrant ASD-risk factor in studies of exquisitely phenotyped nonsyndromic ASD cohorts [11, 26, 27]. Finally, as suggested above, over time, it has become clear that there is considerable overlap in biological mechanisms and molecular pathways implicated in syndromic versus nonsyndromic ASD. As early as the 1990s, gene discovery in monogenic syndromes highlighted the potential contribution of RNA-binding proteins [16, 17, 28–31], synaptic function [32], the mTOR pathway [19, 20], and chromatin modification [18] to the pathophysiology of ASD. These initial findings are now strongly supported from microarray and next-generation sequencing studies of non-sydromic ASD cohort [33–40].

There are nonetheless important themes being captured by these categorizations—the earliest, pre-genomics era discoveries in ASD resulted from the study of genes on the far end of a distribution of population frequency (low), effect size (high), and reliability of the relationship (strong) between genotype and phenotype. As studies moved toward genes with relatively smaller effects and a less reliable relationship between genotype and phenotype, gene discovery became more difficult and the advent of big data, novel methods, and a change in scientific culture were required to advance the field.

### Gene discovery in nonsyndromic ASD

The first step in the transition from productive studies of syndromic forms of ASD to the larger group of ASD affected individuals was marked by a seminal success in 2003. Jamain et al. performed targeted sequencing in select regions on the X chromosome that had previously been found to carry recurrent de novo deletions in three females with ASD, and identified rare disruptive mutations in two X-linked genes encoding neuroligins (*NLGN3* and *NLGN4*) in affected siblings [33, 41]. The critical contribution of this discovery was not uniformly appreciated at the time, given a widespread preoccupation with candidate gene association studies. However, in retrospect, these were not only among the earliest individual genes associated with nonsyndromic ASD but were a harbinger of findings from -omics studies a decade later—including the contribution of both structural and sequence variation, the central role of de novo germline disruptive mutations, the high level of phenotypic variability associated with mutations in the same gene, and the observation of a possible female protective effect.

### Copy number variation studies

It was the evolution of high-density microarrays, capable of identifying submicroscopic variation in chromosome structure (known as copy number variation, CNV), across the genome and in large patient cohorts, that presaged a new wave of success in ASD genomics. In 2006–2007, several groups exploited these newly developed tools and found significant enrichment in the rate of de novo germline CNVs, particularly in simplex families with ASD [34, 42, 43]—defined as families in which there is a single affected offspring and both parents are unaffected.

Importantly, these studies focused on germline rather than somatic mutations, given the relative ease of identifying the former and the hypothesis that such mutations could carry large effects. They found that large de novo CNVs clustered in genomic regions [36, 44, 45], suggesting that this did not reflect a nonspecific increase in the liability for mutagenesis in affected individuals. This further established the foundation for the identification of specific risk regions [5, 35, 36, 46–48]. Importantly, the ability to conduct genome-wide screening to identify formerly “invisible” changes in chromosomal structure was accompanied by the development of rigorous statistical methods to assess the significance of recurrent de novo CNVs mapping to the same genomic interval [36].

An ensuing decade of CNV studies characterized by increasingly high-resolution cytogenetic assays, larger patient cohorts, and statistical methods correcting for genome-wide comparisons, led to an era of reproducible findings and highlighted key aspects of ASD allelic architecture. Collectively, these studies found that: (1) a global burden of de novo germline CNVs is associated with ASD [5, 35, 36, 46–48] and are present in 5–10% of affected individuals—compared with <1–2% in unaffected siblings; (2) multiple recurrent de novo CNVs in specific regions are associated with ASD risk [5] (Table 1); (3) females with ASD have an increased burden of de novo CNV variants [5, 36, 47], supporting a female protective effect; (4) genic CNVs (those that disrupt gene-containing regions of the genome) carry the vast majority of risk compared with intergenic CNVs [47]; and (5) CNV risk loci identified in ASD studies are independently associated with a wide range of neurodevelopmental and neuropsychiatric disorders, including epilepsy, intellectual disability, attention deficit hyperactivity disorder, schizophrenia, bipolar disorder, and Tourette disorder [5, 35, 46, 47, 49–51].

**Table 1:** Recurrent de novo CNVs found in Simons Simplex Collection and Autism Genome Project cohorts.

Cytoband	Location (hg19)	De novo SNVs	Del/Dup	FDR	Associated with
1q21.1	chr1:146,467,203−147,801,691	9	1/8	2 × 10^−9^	
2p16.3	chr2:50,145,643−51,259,674	8	7/1	4 × 10^−8^	*NRXN1*
3q29	chr3:195,747,398−196,191,434	4	4/0	0.02	
7q11.23	chr7:72,773,570−74,144,177	5	1/4	0.0008	
15q11.2-13.1	chr15:23,683,783−28,446,765	10	0/10	<1 × 10^−10^	Angelman/Prader-Willi
15q13.2-13.3	chr15:30,943,512−32,515,843	5	3/2	0.0008	
16p11.2	chr16:29,655,864−30,195,048	19	12/7	<1 × 10^−10^	
22q11.21	chr22:18,889,490−21,463,730	8	4/4	1 × 10^−7^	
22q13.33	chr22:51,123,505−51,174,548	4	4/0	0.02	*SHANK3*, Phelan-McDermid

Adapted from Sanders et al. [5].

An important consideration when interpreting CNV literature is that current assay methods are not optimally designed to characterize certain categories of CNVs, including those that contain highly repetitive content, exhibit a broad copy number range, or are in structurally complex regions of the genome [52–55]. Efforts to address these challenges are ongoing and include employing long-read/third-generation sequencing, increasing read depth, machine learning, and locus-specific droplet-based amplification to obtain more precise copy number characterization [52, 56–58]. These efforts will become more precise as assay methods and statistical approaches are continually refined, and with such it is likely that additional ASD-associated loci will be discovered.

### Whole-exome sequencing

The rapid evolution of genomic technology in the first decade of the 2000s created the preconditions for a transition from the study of submicroscopic chromosomal segments—involving thousands to tens of thousands of base pairs—to high-throughput sequencing at single-base resolution. In its initial implementation, this involved assaying germline sequence variation in nearly all of the coding region, or ~1%, of the human genome. In 2011–2012, four research groups applied this technology, often referred to as whole-exome sequence (WES), to simplex cohorts. Essentially simultaneously, they all found a statistically significant excess of de novo, germline putative loss-of-function mutations—those leading to stop codons, canonical splice site mutations or frameshifts—in ASD probands [11, 27, 38, 39, 59]. These studies addressed the association of specific variants by evaluating the recurrence of de novo damaging mutations at the same locus. Three genes, *SCN2A*, *GRIN2B*, and *CHD8* were found within and across these studies to show significant evidence for ASD risk. The studies also found that there was no increase in the rate of multiple de novo germline point mutations in affected versus unaffected individuals [11], suggesting that a single “hit” was responsible for the observed risk in probands. Interestingly, the rate of de novo mutation was also found to be associated with paternal age, and the vast majority of mutations were traced to the paternal chromosome [39]. Finally, a statistically significant excess of missense de novo mutations was identified [11], a finding that has been replicated in larger cohorts, with an overall effect size less than for putative loss-of-function mutations.

The initial WES studies reported on a combined total of 752 families from the Simons Simplex Collection (SSC) [59] and 175 families from the Boston Autism Consortium. While these numbers are larger than those typically associated with pre-genomic era studies, they are far smaller than the tens to hundreds of thousands of cases and controls typically needed for successful genome-wide association studies (GWAS) (see below). This discrepancy in part derives from the challenges these methods face with regard to statistical power: for GWAS, the combination of very small effects of common alleles, combined with the comparison of hundreds of thousands of loci simultaneously, has required very large patient cohorts to achieve genome-wide significance levels and reproducible results [43, 60–64]. For the early CNV and WES studies in ASD, the effect sizes of the respective mutation types turned out to be substantially larger than those associated with common alleles and the multiple comparison problem more modest. Instead, the major challenge for these investigations has been the low frequency of the variants in question.

Novel statistical approaches have been utilized to overcome power limitations posed by the rarity of germline de novo events. A key insight has been the value of quantifying recurrence of germline de novo mutations at specific genes and regions as opposed to simply counting and comparing numbers in cases versus controls [11, 27, 38, 39]. Given the low number of de novo germline mutations in any individual—sequencing studies have found on average less than 100 de novo germline mutations per human genome—and the associated low frequency at which these are introduced (on average only a single-coding germline SNV per individual per generation), the likelihood of observing by chance multiple “repeats” of any loss-of-function de novo mutation in the same gene or CNV interval, in cohorts of the size studied in ASD, is quite low. Moreover, factors that influence the rate of mutation and likelihood of recurrence for CNVs and SNVs are now well understood. The rate at which multiple damaging de novo mutations map to the same interval or gene in affected individuals, and a comparison either to controls or to expectation, yields a surprising amount of statistical power from a small number of events. More recent statistical developments have included the “transmitted and de novo association” (TADA) method [65, 66] which incorporates information from multiple variant classes, leveraging both the statistical power of rare recurrent de novo germline mutations and the greater frequency of transmitted variants to elegantly maximize the yield of gene identification.

A caveat of the TADA method is that is does not incorporate annotation information and handles only certain categories of de novo mutations, making extension of the technique to noncoding regions challenging. Various approaches have been used to overcome this, including FitDNM, which explicitly incorporates functional information [67] and TADA-Annotations (TADA-A), an extension of the original TADA design, which incorporates functional annotations of noncoding regions and thus can be extended to WGS analyses [68]. Many commonly used methods also use linear models to predict mutational effects, whereas nonlinear models that can incorporate more complex relationships may prove more powerful in predicting variant impact [69]. In addition to the myriad statistical modeling approaches used to identify risk variants, multiple scoring systems to estimate variant deleteriousness have been developed (often incorporated into the above approaches) to optimize detection of deleterious variants [66, 70–72] (Box 1).

Over time, WES studies in increasingly large cohorts have replicated initial findings and dramatically increased the number of genes associated with ASD [12, 40]. Sanders et al. combined WES data from Autism Sequencing Consortium and the SSC cohorts, and, using a version of TADA that combines information from both structural (CNV) and sequence (SNV) findings, identified a total of 71 significant risk loci, including 65 genes and 6 CNVs [5]. The largest WES to date analyzed 21,219 family-based samples and 14,365 case–control samples, identifying 102 significant ASD-risk genes (Table 2), 30 of which have not previously been implicated in ASD or other autosomal dominant neurodevelopmental disorders such as epilepsy, and intellectual disability [4].

**Table 2:** Statistical evidence for association of ASD genes based on rare de novo transmitted sequence variation and de novo CNVs.

FDR ≤ 0.01	0.01 < FDR ≤ 0.05	0.05 < FDR ≤ 0.1
ADNP^a^, ANK2^a^, ANKRD11, AP2S1, ARID1B^a^, ASH1L^a^, BCL11A^b^, CHD2^a^, CHD8^a^, CTNNB1, DEAF1, DNMT3A^b^, DPYSL2, DSCAM^a^, DYNC1H1, DYRK1A^a^, FOXP1^b^, GABRB3^b^, GIGYF1^b^, GRIN2B^a^, KCNQ3, KDM5B ^a^, KDM6B^b^, KMT2C^a^, MAP1A, MBD5^c^, MED13L, MKX, MYT1L^b^, NRXN1^a^, PAX5, POGZ^a^, PTEN^a^, RAI1, RORB, SCN2A^a^, SETD5^a^, SHANK2^a^, SHANK3^a^, SIN3A, SLC6A1^b^, SRPR, SUV420H1^a^, SYNGAP1^a^, TBL1XR1, TLK2, WAC^a^	ASXL3, CACNA1E, CELF4, CREBBP, EIF3G, FOXP2, GFAP, GNAI1, IRF2BPL^c^, KIAA0232, LDB1, NSD1, PHF12^b^, PHF2, PHF21A, PPP2R5D, PRR12, RFX3, SATB1, SKI, SMARCC2, SPAST^b^, STXBP1, TBR1^a^, TCF20, TCF4, TCF7L2^a^, TM9SF4, TRIP12^a^, VEZF1, ZMYND8	CACNA2D3, CORO1A, DIP2A^c^, ELAVL3, GABRB2, GRIA2, HDLBP, HECTD4, KCNMA1, KMT2E^c^, LRRC4C, NACC1, NCOA1, NR3C2, NUP155, PPP1R9B, PPP5C, PTK7^c^, SCN1A, TAOK1, TEK, TRAF7, TRIM23, UBR1

Genes found to be significantly associated with ASD in Satterstrom et al. [4]. Comparison with genes identified by Sanders et al. is indicated (^a^FDR ≤ 0.01 in Sanders et al. [5], ^b^0.01 < FDR ≤ 0.05 in Sanders et al. [5], ^c^0.05 < FDR ≤ 0.1 in Sanders et al.) [5].

Overall, WES studies have demonstrated that the contribution of de novo mutations to ASD is considerable. High-confidence de novo variants increase risk, on average, by 20-fold [12]. Recent studies have variably estimated that 10% of ASD patients carry a contributory single-nucleotide variant (SNV) or CNV (16.6% of girls) [5] and >30% of ASD patients harbor a contributing de novo LoF or missense mutation [73]. Predictive modeling based on these large-scale sequencing studies consistently conclude that hundreds to over 1000 genes will ultimately be found to contribute to autism risk through a vulnerability to de novo germline damaging SNVs [12, 27, 38, 39].

Box 1 Estimating deleteriousness With the expanding wealth of information provided by large-scale sequencing projects, it has become increasingly necessary to develop techniques to reduce noise and improve signal detection when attempting to identify variants associated with disease phenotypes. One focus of these efforts has been on developing scoring systems to estimate the likelihood that a given variant is deleterious. These scoring systems incorporate multiple lines of evidence, such as evolutionary features of the gene in which a suspect variant occurs, the frequency of said variant across different control databases, the impact of the variant on gene function (such as whether it has a protein-truncating effect), and whether it appears to be highly regulated based on epigenetic signatures. These scores are then used to narrow larger candidate lists to improve detection of risk-associated variants. One of the most popular gene-scoring systems in current practice is the probability of intolerance to loss-of-function (pLI) score. This is estimated using the frequency of protein-truncating variants observed in a given gene across multiple control reference databases, accounting for gene size and sequencing coverage. A higher score indicates the gene is more intolerant of loss-of-function mutations, with a cutoff of pLI > 0.9 (or higher) typically used to define likely deleterious variants. Another commonly used score is the Combined Annotation-Dependent Depletion (CADD) score, which ranks deleteriousness of variants (SNVs and indels) in the human reference assembly based on a variety of genomic features including evolutionary constraints, epigenetic features, and sequence context, and is intentionally not trained on variants with known pathogenic/benign status. The Missense badness, Polyphen2 and Constraint (MPC) score, meanwhile, incorporates information on missense-constrained regions within genes to score deleteriousness of missense variants that produce amino acid changes in highly constrained regions. Finally, the loss-of-function observed/expected upper bound fraction (LOEUF) score estimates gene tolerance to loss of function similar to pLI, but is more easily used as a continuous rather than dichotomous value. Each of these approaches (as well as the many others beyond the scope of this review) has unique strengths and weakness that should be considered when incorporating them into any ranking algorithm, including incorporation of evolutionary versus within-population constraints, variant types to which a score can be applied, use of functional information and regulatory features, and application to coding versus noncoding regions.

### Whole-genome sequencing

Whole-genome sequencing (WGS) enables investigation of the vast majority of the sequence of the human genome, including noncoding segments, and is capable of detecting all classes of genetic variation [74]. It has more uniform coverage that microarray or WES and captures variants in the coding region that may be missed by WES [75, 76], suggesting that even if one is only examining the coding region of the genome, WGS is a more sensitive sequencing modality, albeit with a higher cost [75–77]. Thus, as the price of WGS continues to fall and throughput increases, there is little question that it will supplant WES for all high-throughput genomic studies.

Early ASD WGS studies involved cohorts of insufficient size to discern statistically significant association of specific loci in the noncoding genome [78–80]. With WGS, there are two orders of magnitude more sites to consider (~3 billion) compared with potential GWAS loci (functionally ~1 million) or WES variants (~30 million). While larger sample sizes can overcome this barrier, a major ongoing challenge in using WGS is the difficulty in interpreting variation in the noncoding genome [74, 81], which is not well annotated as its functions are not yet well understood. Importantly, it is not entirely clear which noncoding elements in which specific contexts have functional impacts and, even for those motifs where functional properties are better understood, how to predict the impact of specific variants on such function.

Creative approaches have been utilized to overcome these challenges in gene discovery studies. Werling et al. developed an analytical framework for WGS termed category-wide association study (CWAS), which mirrors the statistical rigor of GWAS, with annotation categories in place of SNPs [82]. In CWAS, thousands of categories are defined by combining groups of broad annotations (for example, variants impacting H3K4 epigenetic markers in promotor regions of ASD-associated genes). Each of these categories was tested for enrichment of de novo germline variants in ASD cases versus controls. Applying CWAS to 519 SSC families, no rare noncoding variant categories reached significance [82]. The authors leveraged their empirical results to estimate that over 8000 families would be necessary to identify a category-wide signal in a CWAS of ASD and provided evidence that the contribution of noncoding mutations to ASD risk is modest compared with that of coding variation.

Other studies have attempted to address these challenges using available knowledge of the genome to predict a priori the types of variants most likely to mediate risk, and then focus analysis on those targets (e.g., 5′- or 3′-untranslated regions or putative fetal brain promoters) [75–79, 82, 83]. There is no consensus, however, regarding which noncoding variant types would be highest yield for analysis, leading to a lack of consistent designs across studies. This work has thus not yielded a reliable and reproducible association between de novo noncoding variation and ASD [75–79, 82, 83]. Moreover, these approaches are controversial, as historical attempts to use biological hypothesis to undergird “candidate gene” studies proved to be a fundamentally flawed approach to genetic association [84, 85]. Consequently, efforts to predict and test a set of candidate motifs based on biological plausibility have a very high bar to cross in demonstrating greater consistency and reproducibility than the prior genic version of this same strategy.

In contrast, there is little question that a combination of increased cohort sizes and reliance on hypothesis-free approaches with rigorous correction for multiple comparisons will lead to success in time. This approach will be aided by an increasingly comprehensive annotation of the genome, including the clarification of functional impacts of diverse noncoding elements and accurate assembly of complex coding and noncoding regions—empowered by novel technologies such as long-read single-molecule sequencing that are able to characterize complex regions which have eluded assembly by traditional sequencing approaches [58, 86].

### SNP-based genome-wide association studies

Single-nucleotide polymorphism (SNP)-based genome-wide association studies (GWAS) have significantly advanced the understanding of the contribution of common variation to common human disorders [87, 88]. These GWAS compare SNPs with minor allele frequency >1% in the population between cases and controls to test for association with a trait or disorder/disease. Due to the number of SNPs under investigation, a genome-wide statistical significance threshold of 5 × 10^−8^ is employed to correct for ~1 million independent tests [89]. This, along with the consensus requirement that significant alleles found in an initial screen must be confirmed in independent samples, has resulted in highly reliable results from GWAS across all of medicine, including psychiatry [90, 91].

Despite cohorts consisting of more than 1500 cases, early GWAS in ASD proved to be underpowered [43, 61–64]. However, large-scale international collaborations have repeatedly demonstrated that combining genotyping data across cohorts can improve statistical power [92], and this has proven a successful approach in a wide range of psychiatric disorders [92, 93]. Application of this strategy to ASD has now yielded genome-wide significant results[94]. A recent ASD GWAS examining 18,391 ASD and 27,969 controls, more than twice the size of the largest previous studies, identified five common genetic variants reaching genome-wide significance [60] (Table 3).

**Table 3:** Genome-wide significant loci from ASD scans.

Index variant	Chr	BP	*P*	*β*	s.e.	A1/A2	Freq	Nearest genes
rs910805	20	21248116	2.04 × 10^−9^	–0.096	0.016	A/G	0.76	*KIZ*, *XRN2*, *NKX2-2*, *NKX2-4*
rs10099100	8	10576775	1.07 × 10^−8^	0.084	0.015	C/G	0.331	*C8orf74*, *SOX7, PINX1*
rs201910565	1	96561801	2.48 × 10^−8^	–0.077	0.014	A/AT	0.689	*LOC102723661*, *PTBP2*
rs71190156	20	14836243	2.75 × 10^−8^	–0.078	0.014	GTTTT	0.481	*MACROD2*
rs111931861	7	104744219	3.53 × 10^−8^	–0.216	0.039	A/G	0.966	KMT2E*, *SRPK2*

*Chr* chromosome, *BP* chromosomal position, *A1/A2* alleles, *Freq* allele frequency of A1, *β* estimate of effect with respect to A1; *s.e.* standard error of β, *P* association *P*-value of the index variant (P).

Adapted from Grove et al. [60].

^*^Rare variation in *KMT2E* has been found to be associated with ASD risk with FDR < 0.1 (Sanders et al. [5], Satterstrom et al. [4]). “Nearest genes” lists nearest genes from within 50 kb of the region spanned by all SNPs with *r*^2^ ≥ 0.6 to the index va*r*iant.

Given the early onset of ASD and the significant reduction in fecundity [95], the anticipated effect sizes for individual common alleles carrying ASD risk were smaller than those for many later-onset psychiatric conditions, a prediction that has been borne out by recent empirical evidence. This relative power issue is reflected in the observation that it has required studies an order of magnitude larger in ASD to find the first reliable common alleles versus similar studies in schizophrenia [96, 97]. Given recent successes and the overall maturity of the GWAS field, yields from ASD GWAS are certain to continue to increase as sample sizes increase. Moreover, there are a number of interesting and important questions to be addressed by the integration of common variant and rare variant studies—for example what role common variation plays in determining outcomes for rare highly penetrant CNVs and SNVs.

An increasingly popular approach to leveraging GWAS data relies on polygenic risk scores (PRS). These are used to estimate disease risk based on the cumulative genomic burden of risk variants in an individual [98], summarizing genetic effects by calculating the weighted sum of all associated risk alleles carried by an individual. PRS was first applied in psychiatry in 2009 by the International Schizophrenia Consortium to interpret schizophrenia GWAS findings [97], and PRS design was subsequently developed that predicted case–control status in an independent population [97]. PRS is now a broadly used statistical approach to estimate the genetic influence of markers in GWAS.

While these methods offer promising avenues to investigate a variety of phenomena—from the interaction of common and rare variants in influencing natural history, to predicting treatment response, to informing imaging-genomic studies—there are important considerations in using PRSs in ASD. First, it is essential that the derived scores are applied in extremely well-matched samples as scores are unreliable across divergent ancestral populations [99]. Second, PRSs are by no means diagnostic, or do they predict with certainty that one will or will not develop the disorder (even less so in understudied populations) [99]. Indeed, even high ASD PRS reflects only modest increase in ASD odds, the odds ratio (OR) for individuals in the top versus lowest decile of PRS risk is ~3.6 [62], a small number when compared with ORs of up to ~20 in analogous analyses in schizophrenia [96] and ORs of up to ~15–20 for de novo ASD mutations [12, 100]. Third, PRSs for ASD are associated both with ASD traits in the general population [101, 102], as well as with elevated IQ in unaffected individuals [60], and polygenic risk is often shared among neuropsychiatric disorders (e.g., polygenic risk score for ADHD is also associated with ASD traits) [103]. One area where PRSs may be most useful is in identifying genetic subgroups of those with ASD that may be more tractable for studies of natural history, neuroimaging, or differential response to treatment, although this has yet to be fully developed [60].

## The challenges of translating genes to pathobiology

Over the past decade, discovery efforts in nonsyndromic ASD have been a resounding success, thanks to the evolution of large-scale omics datasets and analyses, generating an increasingly large set of CNVs and protein-altering SNVs that definitively confer risk. However, translating lists of implicated regions and genes into an actionable understanding of biology remains a major challenge for the field.

There are several key hurdles to elucidating core pathologic mechanisms underlying ASD [104]. First, the nature of the offending variant has a major impact on the tractability of the neurobiological question(s). For example, the most common ASD-associated risk CNVs typically involve intervals containing multiple genes, and the range of phenotypic outcomes resulting from a specific CNV is extraordinarily broad. Moreover, there is evidence that especially larger common risk CNVs, such as at 16p11.2, are relatively depleted for de novo damaging mutations [5], suggesting they contribute to ASD via oligogenic or polygenic mechanisms [105, 106]. Consequently, the task of dissecting the relevant contribution of any single gene within these multigenic intervals, specifically with regard to its impact on social functioning, is likely to be particularly challenging.

The evolution and success of WES in ASD is a welcome development, offering a long and growing list of mutations pointing to single genes that impart large effects in a given individual [11]. While the outcomes of these rare de novo damaging germline mutations often include both ASD and ID, there are recent data pointing to a subset that are relatively more specific for ASD [4].

Any of these genes can plausibly be studied individually to elucidate their mechanisms of action. However, more than 100 risk genes have already been identified, and modeling suggests there are several hundred to 1000 gene “targets” of de novo damaging mutations still to be discovered [107]. This locus heterogeneity raises the question whether illuminating the putative function of any individual gene will yield broader insights into ASD pathology.

To complicate matters further, ASD genes have numerous context-dependent biological effects (pleiotropy), which manifest at various levels of organization of the human brain. These effects likely have variable penetrance and expressivity, with dosage effects further contributing to phenotypic variability. Finally, the human brain is an intricate, relatively poorly understood, and largely inaccessible organ composed of diverse, developmentally dynamic cell types underlying a complex array of developmentally influenced circuits. In short, our current understanding of human brain organization at the molecular, cellular, and circuit levels remain strikingly incomplete, limiting the ability to contextualize the role of any given gene.

In the face of these challenges, the traditional approach of studying single large-effect risk genes in model organisms one at a time is not well suited to differentiate potentially myriad, developmentally dependent biological effects from key pathophysiological mechanisms relevant to the human. As is amply demonstrated throughout the neuroscience literature, it is relatively straightforward to identify biological consequences arising from the recapitulation of a human ASD mutation in a model system. It is manifestly more difficult to determine how any such observation at the molecular, cellular or circuit-level relates to the emergence or maintenance of specific features of social disability.

The impulse to address this question through anthropomorphizing behavioral phenotypes seen in mouse and other more evolutionarily distant model systems is commonplace. Investigators often feel bound to search for evidence of changes in an organism’s social behavior that appear similar to human symptoms and, further, to equate the rescue of such phenotypes with evidence for a specific role in ASD pathology. At the same time, the profound differences in the structure and development of, for example, mouse and human brain, including in anatomic regions thought highly relevant for ASD, are widely accepted. It is understood that the core features of ASD are behaviors that are distinctly, and in some cases, uniquely human. The empirical evidence is not reassuring: the track record of relying on face validity in psychiatric disorders including for therapeutics development has, for the most part, been dismal. As increasing volumes of cell-type-specific expression and regulatory data are available across species, the differences at these levels of organization become clearer as does their potential to complicate or even thwart efforts to translate specific findings from a single mutation from one species to another. Thus the field has increasingly relied on an expanded collection of model systems in an effort to move “closer” to humans, including development of brain organoids, patient- and control-derived iPSCs, as well as non-human primate models, the latter of which raises both scientific and ethical questions that are the subject of intense and important ongoing debate.

A full discussion of model systems both in current use and being considered for future exploration is beyond the scope of this discussion, but the selective use of multiple systems in a coordinated fashion, alongside improvements in spatiotemporal resolution of human brain development via imaging and expression assays, holds promise for scaffolding findings to develop and test actionable hypotheses regarding ASD pathology.

### Convergence and systems biology approaches

**Fig. 3:**
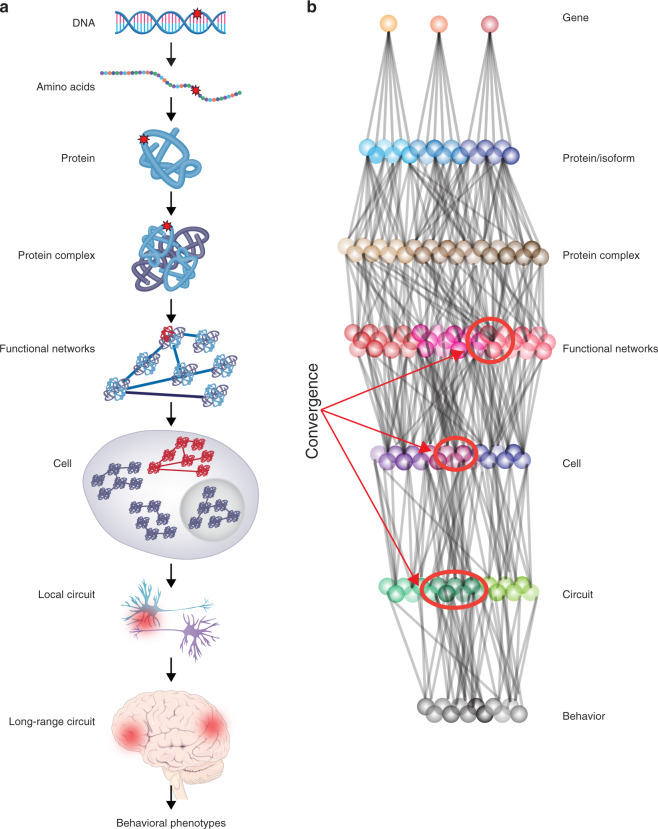
Levels of pathogenesis and convergent analysis. **a** ASD can manifest or be investigated at multiple different levels, starting from a genetic variant (marked by red star) all the way to behavioral phenotypes. **b** A conceptual illustration of convergent analysis from risk genes to behavior in ASD, in which multiple independent risk genes are studied in parallel to triangulate on specific protein complexes, functional networks, cell types, and or/circuits that show overlap among functionally diverse risk genes. Figures adapted from Willsey et al. [13] and Sestan and State 2018 [156] with author permission.

The combination of extensive genetic heterogeneity and tremendous biological pleiotropy coupled with the obvious complexity of human brain development has resulted in the adoption of a number of big-data strategies that compliment or supplant traditional single-gene studies in vitro or in common models. An increasingly promising approach has been enabled by the availability of foundational big-data -omics resources and the systematic discovery of large numbers of ASD-risk genes. This is the search for convergence, based on the notion that at some point in the continuum between genetic mutation and complex human behavior, subsets of ASD-risk genes must intersect to result in a characteristic behavioral phenotype (Fig. 3). These points of intersection may indicate core, conserved aspects of ASD pathology.

Investigations of convergence at the molecular level accompanied the first WES studies [11, 27] highlighting the contribution of synaptic proteins and chromatin modification. Indeed, Huda Zoghbi raised the prospect of convergence at the synapse in 2003, coincident with the discovery of mutations in the genes *NLGN4* and NLGN3 [108].

Multiple subsequent efforts to identify convergence have involved querying larger and larger gene lists against pre-existing biological databases to determine whether specific characteristics are overrepresented. Gene ontology and protein–protein interaction (PPI) analyses have consistently implicated a number of biological functions and pathways, including, but not limited to, chromatin/transcriptional regulation, neuronal development, synaptic function, Wnt/beta-catenin signaling, and Fragile X Mental Retardation Protein (FMRP) targets [5, 12, 26, 38, 47, 77].

**Fig. 4:**
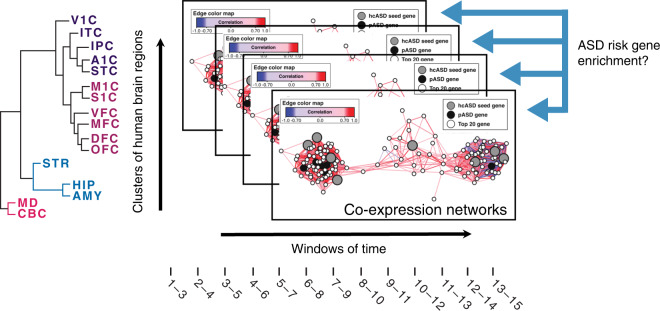
A strategy for combining human brain expression data and high-confidence risk genes to identify spatiotemporal convergence. Willsey et al. [112] established co-expression networks for the nine highest confidence ASD-risk genes at the time of publication. There networks were established by setting a high threshold for gene expression correlation irrespective of sign—based on the hypothesis that coordinated gene activity, whether in the same or opposite directions, is a useful proxy for shared biological function. Networks were created for spatiotemporal periods defined in the Brainspan database [113], using their time windows. Co-expression networks based on the highest confidence genes were then examined for enrichment of an independent list of probable ASD-risk genes and compared to the null expectation, looking for enrichment of genes that have evidence for ASD risk within any of the predefined networks. In this case, statistically significant evidence was found for enrichment of PFC in mid-fetal development at approximately 18–24 weeks, and additional signal was identified in medial dorsal thalamus and cerebellum later in development (in early infancy).

However, while these findings highlight broad molecular pathways and functions of interest, they are “static,” assuming that functional deficits are stable and persistent across developmental time, which may not be true in ASD or other developmental disorders. These concerns, coupled with the opportunity afforded by a growing list of reliable risk genes, prompted several groups to begin working on identifying spatial and temporal convergence in ASD [109–112]. Two of the earliest efforts leveraged data from BrainSpan developmental transcriptome project, which generated gene expression data from early fetal to late adult stages across multiple distinct anatomical regions in 57 typically developing human brains [113] to look for enrichment of ASD-risk genes within brain gene co-expression networks (Fig. 4). While the gene lists and approaches to network development differed somewhat between studies, two simultaneous papers identified human mid-fetal cortical excitatory neurons as an important point of intersection [109, 113]. They differed in findings regarding the specific cortical layers showing greatest enrichment. Both suggested that there would likely be multiple cell types and developmental epochs identified as gene lists expanded and transcriptional databases became more detailed and comprehensive. In addition, in a recent GWAS study, SNPs associated with ASD mapped to genes that are expressed in the developing fetal cortex [60], suggesting that common and rare variants may functionally converge on specific cell types and/or developmental stages.

These findings point to important opportunities to further illuminate ASD pathology. The ability to connect ASD mutations with specific cell types and development time points in human can help guide future experiments looking for pathophysiological phenotypes in model systems. Moreover, the identification of co-expression or protein networks implicated in ASD offers the opportunity to assess how close various model systems come at a molecular level to recapitulating the human context.

These types of approaches promise to become increasingly valuable as reliable gene lists expand and as multidimensional datasets emerge cataloguing the molecular, cellular and regulatory landscape of human brain development and that of other species at greater depth and resolution. The PsychENCODE Consortium, a NIMH effort founded in 2015, represents the largest integrated collaborative effort in neuroscience and psychiatry to collectively analyze genomic regulatory elements in a large cohort of well-curated human brains [115]. PsychENCODE aims to generate a repository of multidimensional genomic data using tissue- and cell-type-specific samples from approximately 1000 phenotypically well-characterized, healthy and disease-affected human post-mortem brains, with an initial focus on ASD, bipolar disorder, and schizophrenia. This will enable comprehensive analyses of regulatory regions, epigenetic modifications, and gene expression patterns across different ages, regions, and cell types in both healthy and disease-affected human CNS. The PsychENCODE project has an additional goal of functionally characterizing disease-associated regulatory elements and variants in model systems (including iPSC and mouse models) [115]. The Allen Institute for Brain Science, founded in 2001, has several ongoing efforts (https://portal.brain-map.org), including the Allen Mouse Brain Atlas [116], which now includes electrophysiological, morphological, and transcriptomic data obtained from individual cells [117, 118], and the Human Brain Atlas, with parallel efforts in profiling human brain cells [114]. BrainVar [119], a comprehensive repository of WGS paired with RNAseq from the human prefrontal cortex of neurotypical individuals across multiple stages of fetal to adult development, will tremendously aid future studies.

Further, early single-cell RNA sequencing (scRNAseq) studies have demonstrated the power of single-cell transcriptomics to provide a framework for understanding the complexity and heterogeneity of cell types in the mouse nervous system [118, 120–124] and developing human brains [125–127]. Advances in scRNAseq technology have enabled higher-throughput studies, with analysis of many more cells to complement and extend prior studies [117, 128–130]. A high-resolution single-cell atlas composed of 40,000 cells from fetal brain tissue identified several cell types, including deep and upper-layer developing glutamatergic neurons, with enriched expression of high-confidence ASD-risk genes [129]. A recent investigation using single-nucleus RNAseq (snRNAseq) to profile brain tissue from 15 ASD and 16 control individuals revealed 510 differentially expressed genes, predominantly in upper-layer excitatory neurons and microglia [131]. These implicated cell types have shared developmental lineages with cell types that were previously implicated in ASD during fetal development using hypothesis-free approaches [109, 112]. The emergence of comprehensive single-cell data resources will further empower the search for convergence at the cellular level and among attendant circuits defined by molecular subtypes.

The maturation of scRNAseq technologies has coincided with transformative new methods to profile genetic, epigenetic, spatial, proteomic, and lineage information in individual cells (reviewed by Stuart and Satija [132]). While the majority of these techniques are in early stages of development, a subset have entered mainstream use, and have potential for broadening the understanding of ASD pathobiology. First, new protocols for CRISPR loss-of-function screens enable readout of expression and genetic perturbations in the same single cells [133–136]. Regressing expression (phenotype) versus genotype can provide insights into ASD gene function and epistatic relationships. Second, emerging single-cell ATAC-seq technologies measure chromatin accessibility in single cells [137–139] and can facilitate the identification of noncoding DNA elements, sequence features, and transcription factors that drive gene expression dynamics.

It is now feasible to make a comprehensive brain “parts list”. Efforts to produce a comprehensive brain cell atlas are ongoing [140], and will provide opportunities to identify cell types and genetic programs that are likely involved in ASD pathobiology. Ideally, future cell census surveys might employ multi-omic approaches, combining transcriptomics, epigenomics, and proteomics in single cells. Emerging methods that pair genetic perturbations with expression readout will facilitate the functional characterization of observational data.

## Application to clinical care and therapeutics

Despite the considerable advances noted above, genetic findings have not yet significantly impacted clinical care for individuals with ASD. The mainstay of treatment has been and remains behavioral intervention, with FDA-approved pharmacotherapies limited to a small number of antipsychotics that do not target core social dysfunction [141]. A review of the data regarding efficacy of early interventions or the results of clinical trials of a wide range of psychoactive medications is beyond the scope of this paper (for reviews of the subject, see French and Kennedy [142] and Goel et al. [143]). Here, we will briefly consider the relevance of gene discovery for clinical diagnosis and emerging prospects for directly targeting rare large-effect mutations in ASD.

### Genetic diagnosis

Currently, best practices for the evaluation of children presenting with ASD, with or without intellectual disability, include chromosomal microarray testing, Fragile X testing, karyotyping (if the mother has had 2+ miscarriages), *MECP2* testing (if female, or if male with specific clinical features) [144–146], and many argue for WES as part of standard screening [147, 148]. The specific workup for an individual is influenced both by presentation and discipline-specific recommendations [146–149]. Overall, comprehensive testing is able to identify a likely causative variant in 15–40% of cases depending on the combination of tests used, sex of the proband, clinical phenotype, and family history [144, 147, 150].

The diagnostic utility of genetic testing for the early identification of ASD is hampered by the broad range of phenotypes associated with large-effect mutations characterized in nonsyndromic cohorts. While it is possible to detect an ASD-associated rare de novo structural or sequence variant prior to the age of standard diagnosis, in the absence of symptoms, it is not possible to confidently predict a specific outcome. It is plausible that the combination of genetic testing and early symptom detection, including potential biomarker modalities such as EEG or eye-tracking [151] could markedly lower the age of diagnosis, but currently, combinations of potential biomarkers including genetic status have not been tested rigorously for clinical practice.

More broadly, in cases of nonsyndromic ASD—as opposed to well-characterized Mendelian forms of ASD such as Fragile X, NF, and TSC—genetic testing is currently unlikely to provide guidance regarding natural history, prognosis, intervention. There are circumstances in which a gene discovery might alert a clinician to be vigilant for comorbid pathology, for example for cardiac abnormalities with 22q11.2 deletion. But typically such abnormalities will be detected before ASD is diagnosed. Even the issue of recurrence risk is not straightforward: the presence of a de novo mutation in one offspring does not necessarily indicate that the risk for a second child returns to the population risk. Instead there is evidence that de novo risk mutations are, in many cases, acting against a background of elevated common variant risk in the family. Importantly, there is ongoing research using gene-first strategies [152, 153] aimed at characterizing clinical features among patients who share rare mutations, which offers promise to expand the clinical value of genetic diagnoses in ASD.

Irrespective of the impact on clinical care, genetic testing is still viewed as valuable by many families, who may request testing because it offers a chance to understand more about the etiology of ASD in their child. Increasingly, having a specific genetic diagnosis enables families and individuals to connect with groups that offer support and practical information, and link families to research programs focused on patients with that particular genotype.

### Gene-targeting therapies

A number of different areas of work have converged over the past several years to support the plausibility of developing treatments that target genes and mutations for early onset neurological conditions. The combination of success in rare variant gene discovery in ASD, critical technological advances in manipulating nucleic acids in vivo and practical success in targeting the central nervous system in infants and children, point to the emerging possibility of clinical trials of gene-focused therapies in syndromic ASD and select cases of nonsyndromic ASD with highly penetrant rare, loss-of-function, mutations.

A driving rationale for considering gene-focused therapies in the most severe forms of ASD derives in part from recent progress in the treatment of spinal muscular atrophy (SMA)—a debilitating and progressive neuromuscular disorder caused by mutations in the *SMN1* gene that is the most common genetic cause of infant death. Both the use of gene therapy to introduce functional copies of *SMN1* into the CNS using an AAV vector [154] as well as the use of antisense oligonucleotides to modulate expression of SMN2 to compensate for loss of SMN1 [155] have proven successful in moderating disease course beginning in infancy.

While SMA is an early onset neurodegenerative disorder, these approaches hold promise for use in early onset severe neurodevelopmental disorders: interventions have been tested and successfully employed in the first months of life, repeated intrathecal injections have proven to be safe, as has the use of both ASOs and AAV. Moreover, these treatments have so far demonstrated generalized effects and lasting (to varying degrees) improvement.

Conceptually, there are a number of reasons that targeting the genetic risk is an attractive strategy for ASD. A key challenge to translating genetic findings into therapeutic hypotheses is the biological pleiotropy and developmental dynamism of many ASD-risk genes—as soon as one explores downstream of the mutation itself, the search space for a therapeutic target expands enormously [156]. In contrast, early intervention aimed at the genetic etiology has a clear target in individuals with rare loss-of-function mutations, namely restoring normal protein levels. Moreover, as the large-effect mutations noted above discovered in cohorts of nonsyndromic ASD are typically heterozygous, there is a normal remaining allele that could potentially be leveraged to restore some if not all of the lost function, a strategy conceptually similar to the manipulation of SMN2 splicing to compensate for the loss of SMN1 in SMA.

Several gene-targeting approaches have been applied in animal models of NDDs, including Angelman Syndrome (AS) and *MECP2* duplication syndrome. AS is caused by a loss of the maternally imprinted gene *UBE3A*. Patients with the disorder have intellectual disability, developmental delay, language impairment, and seizures [157]. Expression of the paternal *UBE3A* is silenced by a long noncoding RNA (lncRNA), and thus potential treatment would restore expression of the paternal gene copy. To this end, ASOs have been designed to reduce levels of this lncRNA. In mouse models of the disease, this has led to improvement in cognitive deficits associated with the disorder, as well as body weight normalization [158]. Notably, not all phenotypes were reversed, and rescuing UBE3A embryonically produced better outcomes than doing so after birth [159], raising the critically important question of when intervention will be required for this syndrome and other forms of ID and ASD.

Gene therapy approaches have similarly been employed in models of *MECP2* duplication syndrome—characterized by ID, motor dysfunction, seizures, and early death. ASOs against *Mecp2* reversed multiple disease phenotypes and eliminated seizures in mouse models of the disorder, and corrected MECP2 levels in human lymphoblastoid cells from *MECP2* duplication patients [160]. The use of AAVs, meanwhile, has been demonstrated to rescue *MECP2*-associated phenotypes [161].

It is very likely that the first concrete steps toward gene targeting in ASD will indeed be taken through clinical trials of Mendelian forms of ID and ASD, as a consequence of their clinical severity, the ability to develop trials with an adequate number of patients carrying the relevant mutation(s), the reliability of the mutations manifesting marked developmental impairment, and the progress that has already been made in demonstrating the effective targeting of the biology with therapies, such as ASOs, that do not directly alter the probands’ genetic code.

Of course, the leap from a successful clinical trial in AS, *MECP2* duplication syndrome, Fragile X or other Mendelian ID/ASD syndromes, to conducting similar trials in nonsyndromic ASD will be formidable. The number of potential targets for intervention is limited by attributes noted above—the severity of the predicted outcome, the frequency of the mutation in the population, the reliability of observing a measurable phenotype that is sufficiently impairing to warrant intervention, and the ability to manipulate the underlying biology in a clinically meaningful way. Multiple approaches may aid in overcoming these barriers. Expanding sample sizes to improve identification of recurrent rare variants with large effect may highlight genetic variants most worthy of attention. Identifying points of spatiotemporal and biologic convergence across implicated genes could underscore common pathways or circuits involved in disease pathogenesis. The phenotypic characterization of patient-specific cell lines and organoids, particularly comparison of such among individuals with similar genomic profiles (e.g., via use of PRS), may also be a useful path forward in designing treatments that are both personalized and potentially applicable to a larger group of patients.

Even if these thresholds are met, there are complex ethical and practical considerations surrounding whether and/or when treatment is indicated given potential risks. First, there are limitations as noted in the reliability of diagnostic prediction in ASD-associated rare mutations. Except for a handful of highly penetrant mutations, the range of outcomes from an ASD-associated CNV or SNV is extremely broad. This reality argues for waiting for emergence of ASD/ID symptoms or associated features, such as seizures, before attempting invasive treatment. This is complicated, though, by uncertainty surrounding the therapeutic window for intervention. Given the early onset of ASD and studies that point to the impact of high-effect mutations in mid-fetal human development, it is possible that in utero therapy would be required. However, there is also evidence from a range of rodent models of monogenic syndromes that rescue of core developmental phenotypes can be achieved even into adulthood [160–164]. Consequently, studies aimed at prioritizing genes based on their potential for reversibility in post-natal development, and leveraging diverse model systems to characterize this potential, could be extremely valuable.

There remain critically important questions regarding what outcome measures can and should be used to assess treatment efficacy. Current measures used in clinical trials of ASD are entirely inadequate for the task at hand. Reliably measuring change in social functioning over time and across development remains a daunting challenge for the field. Similarly, at present, there are no validated early biomarkers for ASD in general that are clearly stable, reliable, and capture clinical change—though there is important ongoing work in this area [151, 165–167]. In addition, the likelihood of small numbers of individuals in any clinical trial of this type would render the current strategies of comparing group means to assess ASD treatment efficacy quite challenging. In this regard, it may be that assessment of associated features such as intellectual disability or seizures, as opposed to core social deficits, will be the first metrics used to assess viability of gene-targeting strategies.

Lastly, there are ethical and regulatory considerations to take into account when pursuing gene therapy. Designing and testing treatments for disorders that affect children, particularly treatments that are intentionally designed to impact developmental trajectories, necessitates a comprehensive evaluation of potential risks and benefits.

## Future research directions

The ability to clarify fully the genomic architecture and specific variations contributing to ASD is within reach. With the development of larger patient cohorts and the application of existing methods, relevant genetic risk can be elucidated. This capacity raises a number of important questions. For example, given limited resources, there are certain to be debates regarding the relative merits of extending the long list of ASD genes vulnerable to rare de novo mutations, expanding the far shorter list of common alleles, focusing on the intersection of rare and common alleles in determining natural history or treatment response, exploring the noncoding genome for rare mutations through WGS, and more generally prioritizing the study of diverse populations which are profoundly understudied at present.

However, genetics are a means to an end in the study of ASD and some of the most exciting research opportunities no longer involve gene discovery, but rather have been enabled by these successes. The application of increasingly deep and broad biological databases, and importantly the addition of proteomics datasets to regulatory and transcriptional human brain resources, promises to be profoundly important for systems biological studies in ASD and have the potential to reveal pathobiology and novel targets for treatment.

In addition, the past decade of discovery has opened the door for gene targeting and nucleic acid manipulation as novel treatment modalities sooner in ASD than in any other common psychiatric disorder. These interventions will undoubtedly only be directly applicable to a small minority of patients. However, the impact on individual families could be enormous, and the knowledge gained may provide insights that extend well beyond the subset of those with ASD carrying rare large-effect mutations. A major challenge to moving in this direction, and an obstacle to other efforts aimed at developing rational therapies, is the current limitation in measuring relevant phenotypes and the lack of detailed understanding of developmental trajectories in very young children with ASD [168, 169], including those carrying rare mutations. Consequently, advances in clinical measurement, prospective studies of natural history and development of biomarkers that can be assayed very early in development, are key parts of any ASD research agenda.

## Funding and disclosure

MWS is funded by grants NIH/NIMH U01 MH111662, NIH/NIMH U01 MH116487, and U01 MH115787-01A1. He serves on the scientific advisory board of Blackthorn therapeutics. VBSQ and BW are supported by the National Institute of Mental Health grant R25MH06048.
